# SNPs2ChIP: Latent Factors of ChIP-seq to infer functions of non-coding SNPs

**Published:** 2019

**Authors:** Shankara Anand, Laurynas Kalesinskas, Craig Smail, Yosuke Tanigawa

**Affiliations:** Department of Biomedical Data Science, Stanford University, Stanford, CA 94305, U.S.A.

**Keywords:** non-coding genome, functional interpretation, epigenome, chromatin immunoprecipitation sequencing (ChIP-seq), latent factor discovery, biomedical ontology, enrichment analysis

## Abstract

Genetic variations of the human genome are linked to many disease phenotypes. While whole-genome sequencing and genome-wide association studies (GWAS) have uncovered a number of genotype-phenotype associations, their functional interpretation remains challenging given most single nucleotide polymorphisms (SNPs) fall into the non-coding region of the genome. Advances in chromatin immunoprecipitation sequencing (ChIP-seq) have made large-scale repositories of epigenetic data available, allowing investigation of coordinated mechanisms of epigenetic markers and transcriptional regulation and their influence on biological function. To address this, we propose SNPs2ChIP, a method to infer biological functions of non-coding variants through unsupervised statistical learning methods applied to publicly-available epigenetic datasets. We systematically characterized latent factors by applying singular value decomposition to ChIP-seq tracks of lymphoblastoid cell lines, and annotated the biological function of each latent factor using the genomic region enrichment analysis tool. Using these annotated latent factors as reference, we developed SNPs2ChIP, a pipeline that takes genomic region(s) as an input, identifies the relevant latent factors with quantitative scores, and returns them along with their inferred functions. As a case study, we focused on systemic lupus erythematosus and demonstrated our method’s ability to infer relevant biological function. We systematically applied SNPs2ChIP on publicly available datasets, including known GWAS associations from the GWAS catalogue and ChIP-seq peaks from a previously published study. Our approach to leverage latent patterns across genome-wide epigenetic datasets to infer the biological function will advance understanding of the genetics of human diseases by accelerating the interpretation of non-coding genomes.

## 1. Introduction

Genome-wide association studies (GWAS) have successfully identified many associations between genetic variants and human diseases.^1,2^ However, functional interpretation of these associations remains challenging as most GWAS hits fall into non-coding regions of the genome.^3^ Advancements in high-throughput genome-wide molecular profiling methods, such as ChIP-seq, enable molecular characterization of gene regulatory landscapes, such as histone modification and transcription factor (TF) binding profiles.^4^ Leveraging growing biomedical ontologies, such as the gene ontology (GO), human phenotype ontology (HPO), and Mouse Genome Informatics (MGI) phenotype ontology, tools based on statistical enrichment analysis on genomic regions, such as the genomic region enrichment analysis tool (GREAT), have been used to investigate the function of the non-coding genome.^5–9^ Further, collaborative research efforts, such as ENCODE, the Roadmap Epigenomics project, and GTEx, have also systematically generated data-rich molecular catalogues.^10–12^ These large-scale epigenomic profiles, as well as other publicly available datasets on the NCBI sequence read archive, are integrated into epigenetic data resources, such as ChIP-Atlas and ReMap, which provides an emerging opportunity for data mining and meta-analysis.^13,14^

Advancements in epigenetic analysis suggest that latent patterns in epigenomic regulatory profiles can be discovered and characterized. For example, one TF can bind to numerous genomic loci with specific sequence features and multiple TFs can work together by forming dimers, executing coordinated transcriptional regulatory programs.^15^ Moreover, it is known that many TFs have multiple functions through precise coordination in different contexts, that there are known interactions between histone modifications and TF occupancy, and that histone modifications and TF occupancy influence gene expression.^10–12,15^ With these phenomena in mind, there has been work in harnessing these patterns for functional interpretation of non-coding genomes. ChromHMM, an unsupervised statistical learning method, successfully summarizes patterns of histone modifications as an interpretable annotation, chromatin state,^16^ while eQTL studies examines non-coding variants in light of molecular phenotype, such as expression levels of neighboring genes.^12^ While these approaches show some success in utilizing neighboring epigenomic signals to explore molecular interpretation of non-coding genomes, they are limited in leveraging genome-wide patterns of both histone modification and TF occupancy across different functional contexts. In principle, one can extend these analyses by leveraging all experimentally collected epigenomic profiles and characterizing latent patterns for functional interpretation of non-coding genomic regions on a genome-wide scale.

Here we present SNPs2ChIP, a novel method to infer function of non-coding variants by (1) characterizing latent patterns in epigenomic regulatory profiles using an unsupervised latent factor discovery algorithm applied to 652 ChIP-seq tracks in the ChIP-Atlas dataset, (2) inferring the biological functions of the identified latent factors using GREAT enrichment analysis, and (3) development of a pipeline that takes genomic loci as input and infers functionality of the loci by identifying relevant latent factors using a quantitative score. Our computational approach contributes to dissecting the genetic architecture of human diseases by accelerating functional interpretation of non-coding variants.

## 2. Results

### 2.1. SNPs2ChIP *analysis framework overview*

**Fig. 1.**
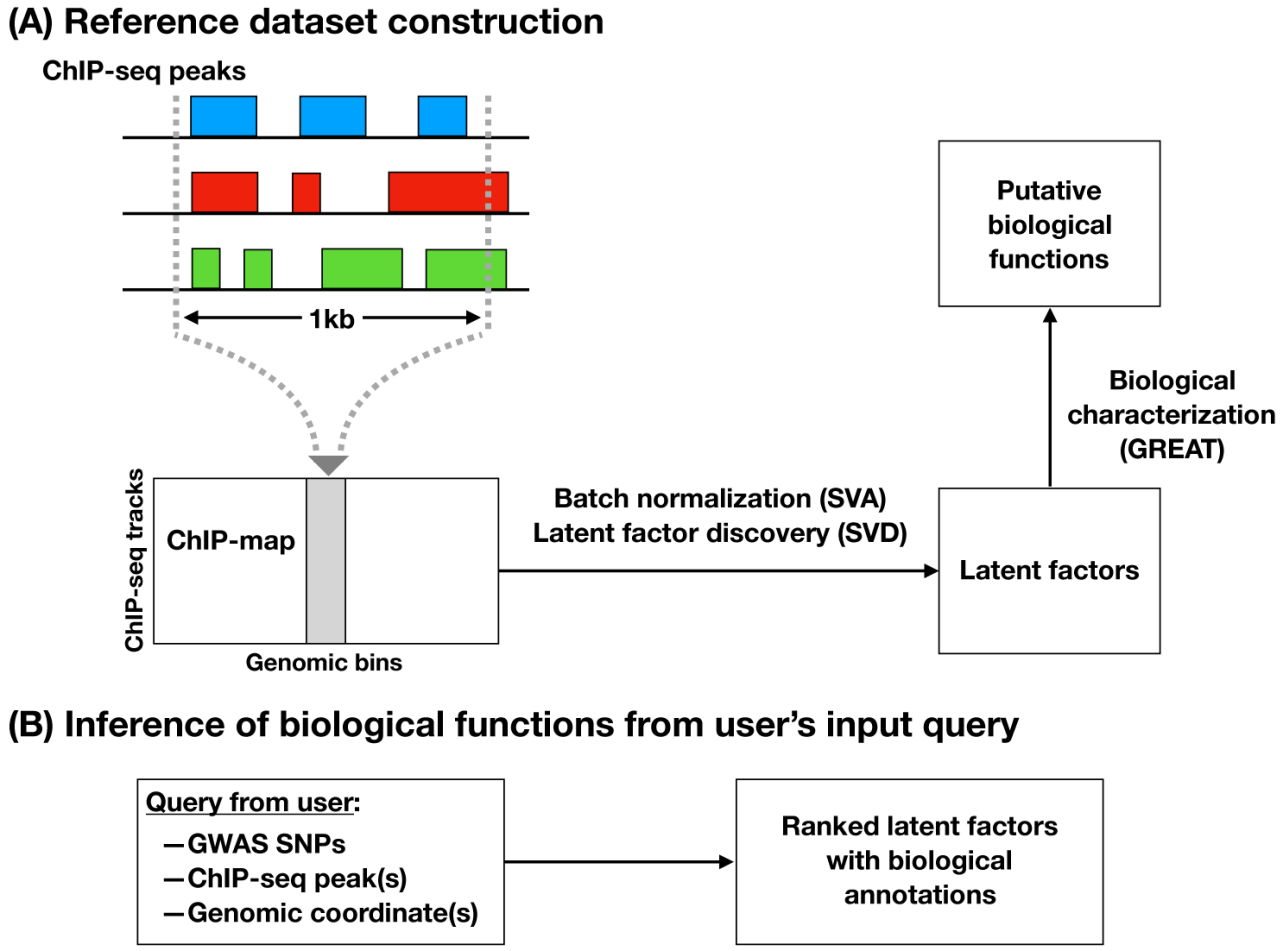
SNPs2ChIP method overview. (A) Construction of SNPs2ChIP reference dataset. ChIP-seq peaks of 652 assays are aggregated into a feature matrix, ChIP-map, followed by batch normalization with surrogate variable analysis (SVA). Latent factors are characterized with singular value decomposition (SVD) and their biological functions are inferred with the genomic region enrichment analysis tool (GREAT). (B) SNPs2ChIP pipeline. Using the pre-computed reference, SNPs2ChIP identifies the most relevant latent factors and returns them with their annotated biological functions.

We developed a method, SNPs2ChIP, to infer functions of non-coding loci that consists of two computational steps: (A) construction of reference ChIP-maps and (B) using the reference ChIP-maps to infer biological functions for user queries. To briefly summarize the first part of our method, we collected chromatin-profiling data from ChIP-Atlas, one of the largest publicly-available databases of ChIP-seq signals with manually curated metadata, and featurized the ChIP-seq peaks across TFs and histone marks into a matrix, called a “ChIP-map.”^13^ To balance the trade-off in specificity of the functional prediction and the genomic coverage of the ChIP-map, we prepared two matrices for high-specificity and high-coverage analysis, by varying the stringency of the featurization methods. After featurization, we applied batch normalization with surrogate variable analysis (SVA) and singular value decomposition (SVD) in each map, resulting a latent factors preserving a linear structure optimal for interpretation..^17^ This was followed by applying GREAT to find the biological functions enriched in each latent factor (Fig. 1A).^9^ With latent factors and enriched functions as a reference, we developed a pipeline that takes a loci as input and returns a list of relevant latent factors as well as their enriched function. A query can be one of many loci: GWAS SNPs, ChIP-seq peaks, or a genomic coordinate of interest (Fig. 1B).

### 2.2. Batch normalization of heterogeneous epigenetic features

We focused on 652 lymphoblastoid cell line experiments, the most numerous cell line in the ChIP-Atlas database, and downloaded all non-empty ChIP-seq peak files. We divided the entire genome into genomic bins of 1 kbp in size and placed ChIP-seq peaks, represented by the strength of the peak, into the bins. This was done across 652 tracks, which created a ChIP-map matrix. After removing genomic bins that did not contain any peaks, we found 379,541 (covering 12.1% of genome) genomic bins and 662,024 (21.1%) genomic bins for the high-specificity dataset and high-coverage dataset, respectively (Methods).

To normalize batch effects in each ChIP-map, we applied the SVA algorithm, a normalization method useful when technical covariates are not known or have missing entries^17^ Out of 39 significant surrogate variables (SVs) identified from SVA, we found that three SVs were significantly associated (p-value < 1.0 × 10^−30^, linear regression) with antibody - a biological effect necessary to protect. The first SV captured variation attributed to H3K4me1 and H3K4me3; the second SV H3K27ac and H3K4me3; and the third SV CTCF, H3K4me3 and SA1. Note that the variation from one sub-group of a given covariate can be split across multiple SVs, as is the case with H3K4me3.

**Fig. 2.**
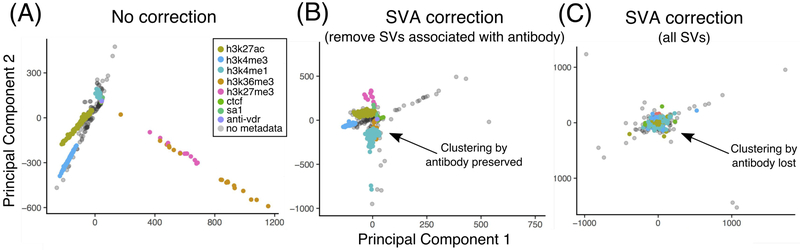
Batch normalization of ChIP-map with SVA.

We assessed the effect of the removing these SVs when regressing out SVs from ChIP-map and compared with results of keeping all SVs in the regression. We implemented the regression using a QR decomposition, enabling an efficient, high-dimensional multivariate multiple regression. When removing SVs significantly associated with antibody, clear clusters were preserved in the corrected data reflective of antibody, but not for technical effects (for example, ancestry). Conversely, when we including all SVs in the regression, no clusters were observed for antibody, indicating an over-correction of data, i.e. removal of biological signal of interest (Fig. 2). Therefore, using a combination of SVA, linear regression and clustering, we were able to preserve biologically important variation while removing unwanted technical variation.

### 2.3. Latent factor discovery and their biological characterization

To find interpretable latent factors in an unbiased manner, we applied an unsupervised statistical learning algorithm, SVD, to the batch normalized ChIP-map. Using the high-specificity dataset, we found that the first three latent factors explain 8.2%, 6.0%, and 4.6% of the variance, respectively, and that the top 50 and 100 factors comprehensively explain 59% and 72.5% of the variance, respectively. For the high-coverage dataset, we found the first three latent factors explain 14.0%, 10.7%, and 5.7% of the variance, respectively, and that the top 50 and 100 factors comprehensively explain 72.6% and 82.6% of the variance, respectively.

To characterize the biological functions of each latent factor, we identified the top 5,000 genomic bins ranked using the genomic bin contribution score derived from decomposed matrices by SVD (Methods - Equation 1). We applied GREAT enrichment analysis for the top genomic bins in each latent factor and identified enriched functional terms using three ontologies: GO, HPO, and MGI phenotype ontology.^5–9^

### 2.4. SNPs2ChIP *identifies relevant functions of the non-coding genome*

To illustrate the utility of SNPs2ChIP to infer the function of non-coding genome, we applied the pipeline to known GWAS SNPs and ChIP-seq peaks from previously published datasets.

#### 2.4.1. Genome-wide SNPs coverage of the reference datasets

**Fig. 3.**
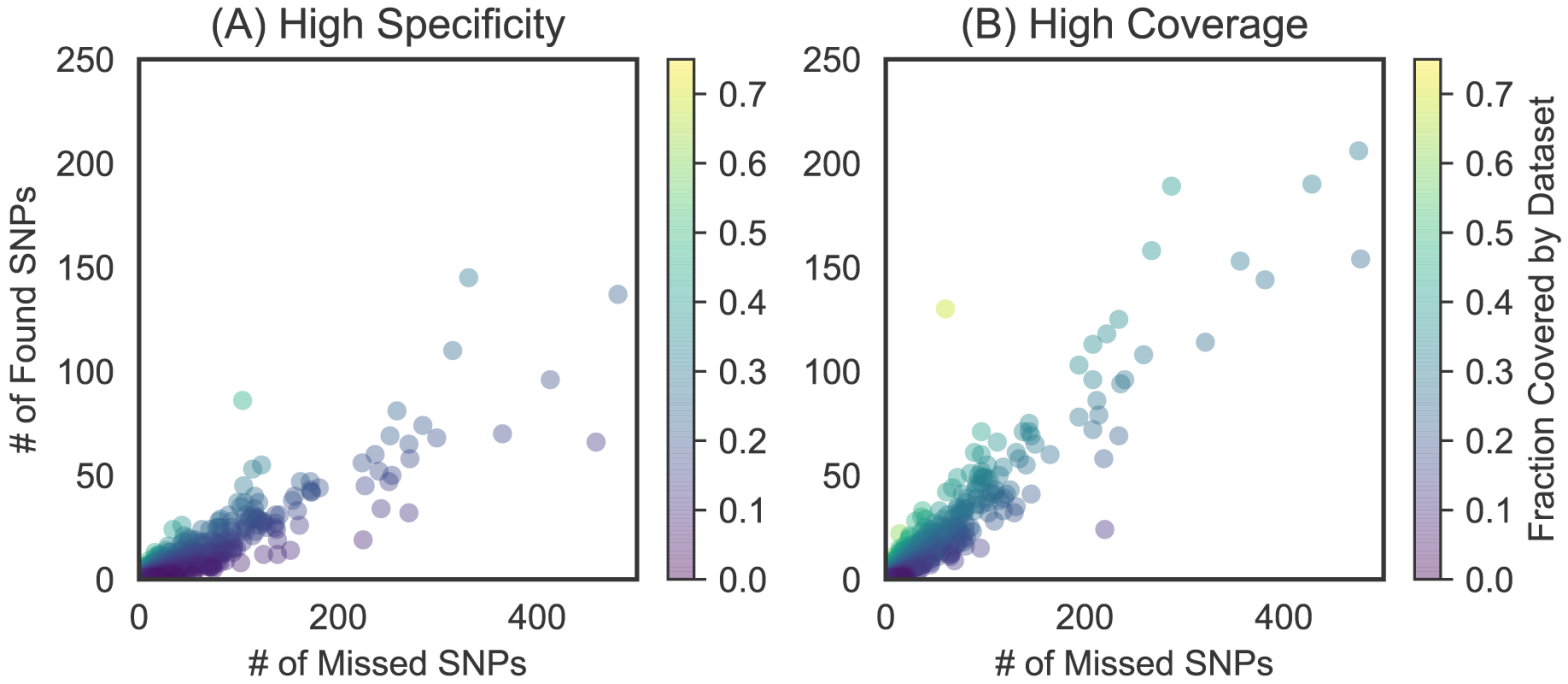
Genome-wide coverage of the reference datasets of SNPs2ChIP. For each disease/trait in the GWAS catalog, we queried SNPs2ChIP and summarized what percentage of the SNPs can be mapped to the relevant latent factors for the (A) high specificity dataset and (B) high coverage dataset.

Given that our reference datasets do not contain empty genomic bins, thus excluding parts of the genome, we first evaluated the coverage of our reference dataset by applying the SNPs2ChIP pipeline to all previously reported SNPs from the GWAS catalogue.^1^ We applied the pipeline for each disease/trait and summarized the number and percentage of SNPs covered by our reference datasets. Out of the 51,892 known non-intergenic GWAS SNPs we tested, we found our high-specificity and high-coverage datasets covers 9,241 (17.8%) and 14,636 (28.2%) of SNPs (Fig. 3).

#### 2.4.2. Non-coding GWAS SNPs of systemic lupus erythematosus

To illustrate the utility of our approach to infer biological functions associated with noncoding GWAS SNPs of diseases, we performed a case-study on systemic lupus erythematosus (SLE). SLE is an autoimmune disorder with a prevalence of 0.1% and a poorly characterized genetic and epigenetic basis.^18^ Out of 425 GWAS SNPs associated with SLE, 110 and 158 SNPs are covered in the high-specificity and high-coverage reference dataset, respectively. Applying the pipeline to the SNPs covered by high-specificity dataset, the top latent factor identified explained 10.7% of the variance in the epigenetic landscape and was enriched for multiple biological concepts associated with SLE. Using HPO as the reference ontology, we found human phenotypes, such as “Abnormality of cells of the lymphoid lineage” (HP:0012140, binomial FDR = 2.7 × 10^−4^), “Lymphopenia” (HP:0001888, FDR = 2.8 × 10^−4^), and “Hemolytic anemia” (HP:0001878, FDR = 2.9 × 10^−4^), which are all known phenotypes for SLE.^19,20^

#### 2.4.3. ChIP-seq peaks for vitamin D receptors

To further test the applicability of SNPs2ChIP, we applied the pipeline to ChIP-seq peaks assosciated with vitamin D receptors (VDR) as an example. Vitamin D is known to participate in transcriptional regulation through VDRs and regulates calcium homeostatic functions.^21^ Its deficiency has been implied in multiple phenotypes, including increased risk of fracture, muscle weakness, and skeletal mineralization defect.^22^

Using the ChIP-seq peaks highlighted in a previously published study,^23^ we applied SNPs2ChIP and identified relevant phenotypes, such as “Parietal foramina” (HP:0002697, FDR = 1.3 × 10^−4^) and “Flat forehead” (HP:0004425, FDR = 2.3 × 10^−3^).

### 2.5. Robustness Analysis in the latent factor identification

**Fig. 5.**
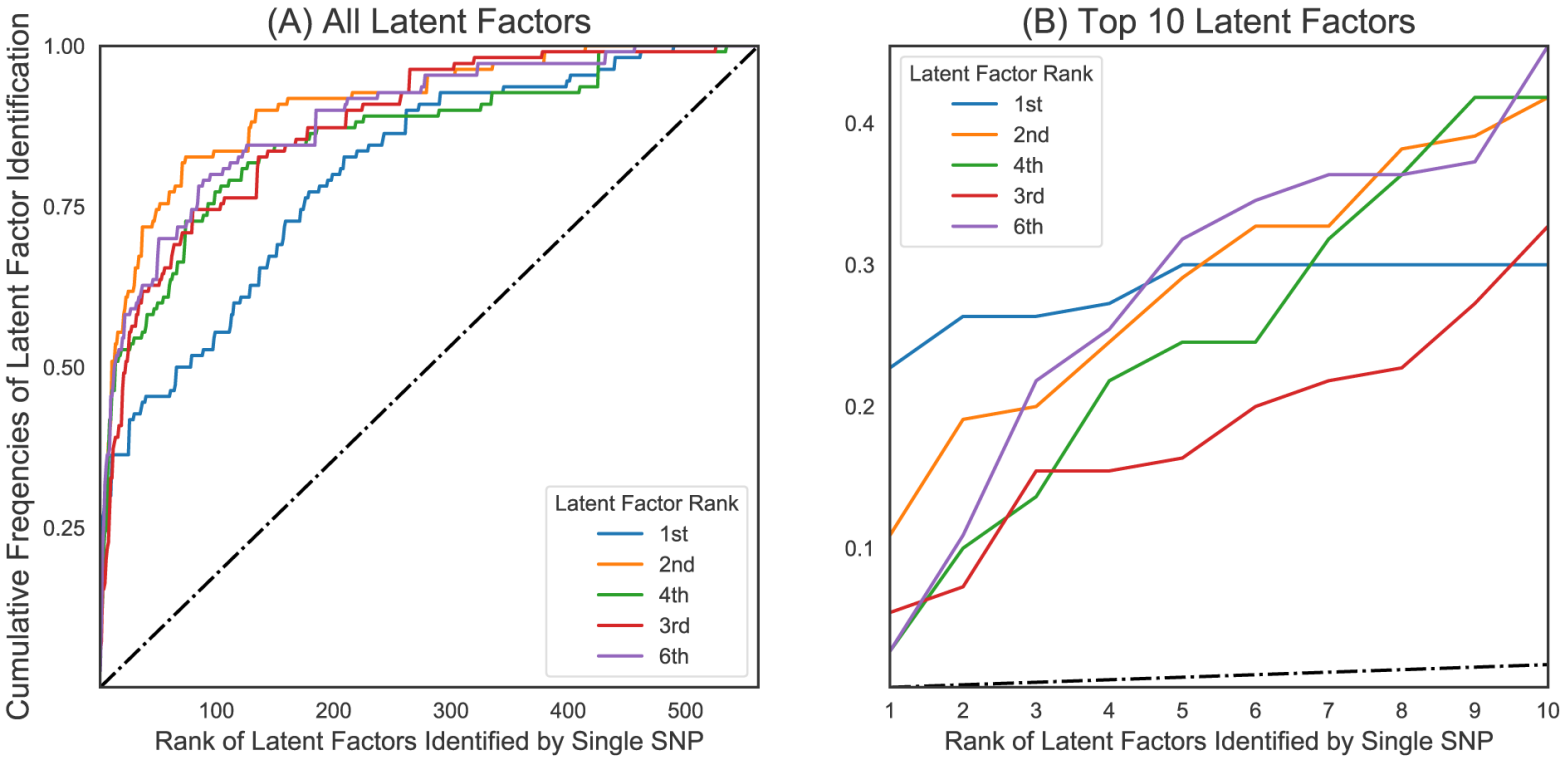
Robustness analysis in the latent factor identification. By using all SNPs associated with SLE, we found the top 5 relevant latent factors (Methods, Eq. (4)). Iterating through each SNP, we plot the cumulative frequency of identifying each of the top 5 latent factors within the rank specified for (A) all latent factors and (B) top 10 ranks. The dashed black line indicates the cumulative frequency under the random null model.

In the SNPs2ChIP pipeline, the identification of the relevant latent factor given a user query is a critical step. To assess the robustness, we applied the pipeline on all of the SLE associated SNPs with the high-specificity dataset and found the top 5 latent factors enriched across the group (Methods, Eq. (4)). We then applied our pipeline on each SNP independently and identified the relevant latent factors for each single SNP (Methods, Eq. (2)). We recorded the number of SNPs that successfully mapped to each of the top 5 latent factors within the top n ranks and reported the results as a cumulative distribution (Fig. 5).

## 3. Discussion

In this study, we propose a new method, SNPs2ChIP, to infer the function of loci in the noncoding genome by leveraging latent patterns in ChIP-seq data from publicly available data. Using latent factors characterized by SVD and annotating them with biomedical ontologies, we developed a pipeline allows us to take genomic regions as input and return relevant latent factors with their enriched biological functions. We applied our method to GWAS SNPs and found that SNPs2ChIP can identify relevant biological functions associated with disease, demonstrating the utility of the genome-wide epigenomic latent factors in the interpretation of non-coding SNPs. In addition, we demonstrated the applicability of our method for vitamin D receptor ChIP-seq peaks, illustrating the utility of our approach for a diverse set of queries.

Further, as shown in our robustness analysis, SNPs2ChIP has an ability to identify relevant latent factors and functions even from a single SNP. This is a major advantage of SNPs2ChIP: it requires a minimal amount of input, one genomic coordinate, to infer biological function as it leverages latent patterns in the epigenome from across the whole genome.

As we rely on existing ChIP-seq data and we focused on lymphoblastoid cell lines, our reference dataset has limited coverage of the genome, which is 12.1% and 21.1% for our high-specificity and high-coverage datasets, respectively. While they still provide a GWAS set coverage of 17.8% and 28.2%, a further expansion of the reference dataset may expand the applicability of the methods.

The resources made available with this study, including the SNPs2ChIP pipeline as well the processed datasets, can provide a starting point to infer the biological functions of noncoding genomes. Combined with the expansion of large-scale epigenomic datasets,^13,14^ our results highlight the utility of latent factor analysis in interpreting the non-coding genome.

## 4. Methods

### 4.1. Featurization of the heterogeneous epigenetic assays

From the ChIP-Atlas database, we downloaded all available ChIP-seq peak files with FDR corrected *q*-value threshold of 1.0 × 10^−5^ for lymphoblastoid cell lines.^13^ Out of the 682 BED files we obtained from the database, we found that 652 were non-empty and used these for our analysis. To featurize the data, we defined genomic bins of size 1kbp across all autosomes and saved them as a custom, genomic bin BED file. For the high-specificity dataset, we kept the top 25,000 statistically significant peaks for each of the 652 BED files, to minimize the confounders due to experimental design, and intersected each of them with the genomic bin BED file using BEDTools.^24^ For the high-coverage dataset, we used all of the peaks in the BED files and intersected these with the genomic bin BED file. For each pair of genomic bin and ChIP-seq assay from the BED intersection, we aggregated the negative log *q*-values into a matrix and removed the genomic bins with no peaks. We generated two ChIP-maps, our feature matrices, for both the high-specificity and high-coverage datasets.

### 4.2. Batch normalization by surrogate variable analysis

We applied the Surrogate Variable Analysis algorithm to the centered, scaled, and log-transformed input ChIP-map to eliminate technical effects which may obscure biological variation.^17^ SVA identifies, in an unsupervised manner, batches of variation across rows and columns of the input data matrix that appear at a frequency greater than expected by chance; each of these batches is represented as a single surrogate variable. We observed that the metadata for the samples had a high rate of missingness; therefore, we devised a novel two-step approach for the removal of technical effects and the protection of biological effects of interest. In the first step, we found statistically significant associations between SVs and known covariates for the set of samples with non-missing metadata using linear regression (where a highly significant p-value indicates high correlation between SV and covariate). As a result, we were able to assign labels to SVs based on the likely biological or technical variation captured by each SV. In the second step, we removed the SVs associated with biological effects of interest, and regressed out the remainder from the input data matrix. We investigated the quality of SVs and the preservation of biological signal through manual inspection of principal component analysis plots.

### 4.3. Latent factor discovery with singular value decomposition (SVD)

We applied SVD for our SVA normalized matrix. The normalized matrix, which we denote as *W*, is of size *N* × *M*, where *N* and *M* denote the number of ChIP-seq tracks and genomic bins, respectively. We obtained the decomposition, *W* = *U DV^T^*, where *D* is a diagonal matrix of size *K* × *K* whose elements are singular values, *U* is an orthonormal matrix of size *N* × *K* whose columns are left (ChIP-seq track) singular vectors, and *V* is an orthonormal matrix of size *M* × *K* whose columns are right (genomic bin) singular vectors. While singular values in *D* represent the magnitude of the latent factors, singular vectors in *U* and *V* summarize the strength of association between latent factors and ChIP-seq tracks, and latent factors and genomic bins, respectively.

#### 4.3.1. Quantification of strength of associations between latent factor and genomic bins

To quantify the strength of associations between latent factor and genomic bins, we define several quantitative scores built on the linear structures of latent factors.^25^ We first define the **factor score matrix for genomic bins** as *G* = *V D*. Mathematically, the factor score matrix is equivalent to the matrix consisting of principal component vectors.^25^ Each element of this matrix, which we call the **genomic bin factor score** and denote as *g_j,k_*, is the projection of the *j*-th column vector in the input matrix *W* of length *N*, which represents the epigenetic landscape of *j*-th genomic bin across samples, to the *k*-th latent factor (principal component).^25^

To quantify the relative importance of a genomic bin for a given latent factor, we define the **genomic bin contribution score** for *k*-th latent factor by squaring the genomic bin factor scores for *k*-th factor and normalizing it across latent factors, i.e.

(1) $${\mathrm{cntr}}_{k}^{\text{bin}}(j)={\left(v_{j,k}\right)}^{2}$$

Because of the denominator in Eq. (1), The sum of genomic bin contribution scores across genomic bins is guaranteed to be one, i.e. $\sum_{j}{\mathrm{cntr}}_{k}^{\text{bin}}(j)=1$, because V is an orthonormal matrix. One can interpret the score as the percent-importance of a genomic bin for the factor.^25, 26^

Similarly, to quantify the relative importance of a latent factor for a given genomic bin, we define the **genomic bin squared cosine score** for *j*-th genomic bin as follows:

(2) $${\cos^{2}}_{j}^{\text{bin}}(k)=\frac{{\left(g_{j,k}\right)}^{2}}{\sum_{k^{\prime }}{\left(g_{j,k^{\prime }}\right)}^{2}}$$

As the sum of genomic bin squared cosine scores across latent factors is guaranteed to be one, i.e. $\sum_{k}\cos_{j}^{2^{\text{bin}}}(k)=1$, we can interpret the score as the relative importance of latent factors for a particular genomic bin.

#### 4.3.2. Quantification of strength of associations between latent factor and samples

We also define the same set of scores to quantify the strength of associations between latent factors and samples. We first define the **factor score matrix for samples** as *S* = *U D* = (*s_i,k_*)*_i,k_*. To quantify the relative importance of samples to latent factors and latent factors to samples, we define the **sample contribution score** and the **sample squared cosine scores** as follows:

(3) $${\mathrm{cntr}}_{k}^{\text{sample}}(i)=\frac{{\left(s_{i,k}\right)}^{2}}{\sum_{i^{\prime }}{\left(s_{i^{\prime },k}\right)}^{2}};\;{\cos^{2}}_{i}^{\text{sample}}(k)=\frac{{\left(s_{i,k}\right)}^{2}}{\sum_{k^{\prime }}{\left(s_{i,k^{\prime }}\right)}^{2}}$$

With these scoring systems we can effectively quantify the associations among latent factors, genomic bins, and samples.

### 4.4. GREAT *analysis for biological characterization of latent factor*

To characterize the functions of latent factors, we applied GREAT version 3.0.0 to each latent factor.^9^ Using ontology-based gene annotations as a reference, GREAT takes a set of genomic regions as an input and reports enriched ontology terms. In our analysis, we focused on gene ontology (GO), human phenotype ontology (HPO), and Mouse Genome Informatics (MGI) phenotype ontology.^5–8^ For each latent factor, we created the query files for GREAT by selecting the top 5,000 genomic bins ranked by genomic bin contribution score (Eq. (1)). We applied GREAT for these queries using default parameters.^9^ Given our interest to characterize the putative functions of non-coding genomes, we focused on the GREAT binomial test and collected summary statistics, such as binomial p-value, binomial FDR, and binomial fold change. We sorted the functional terms outputted by GREAT using binomial FDR and identified the ontology terms that most characterize the function of each latent factor.

### 4.5. *Application of the* SNPs2ChIP *pipeline for GWAS hits and ChIP-seq peaks*

The SNPs2ChIP pipeline consists of three steps: (1) identification of the genomic bins given a user query, (2) identification of the relevant latent factors for the genomic bins, and (3) reporting the results of GREAT enrichment for the relevant latent factors.

#### 4.5.1. Identification of the genomic bin for a given user’s query

SNPs2ChIP takes genomic coordinates as an input. For GWAS SNPs and ChIP-seq peaks, one first needs to obtain their genomic coordinates. These coordinates are then mapped to the corresponding genomic bins, if they contain a ChIP-seq peak.

#### 4.5.2. Identification of the relevant latent factor for the genomic bins

We identify the relevant latent factors for a given genomic bin by genomic bin squared cosine score (Eq. (2)). We can identify the relevant latent factors for multiple genomic bins, which typically corresponds to multiple inputs, by taking a weighted average of genomic bin squared cosine scores. Let’s denote *J* = {*j*_1_,…, *j_m_*} be the set of genomic bins of interest and {*w*_1_,…, *w_m_*} be the corresponding weights. We defined the weighted average of genomic bin squared cosine score as follows:

(4) $${\cos^{2}}_{J}^{\text{bin}}(k)=\frac{\sum_{j\in J}w_{j}\cdot {\cos^{2}}_{j}^{\text{bin}}(k)}{\sum_{j\in J}w_{j}}$$

We set the default value of weights to be uniform, i.e. {*w_1_,…, w_m_*} = {1/*m*,…, 1/*m*} but the user can specify a set of weights based on external knowledge, such as statistical significance and effect size estimates from GWAS. Once we identify the relevant latent factors, we report the results of GREAT enrichment analysis to the users.

#### 4.5.3. *Systematic application of* SNPs2ChIP *for known GWAS hits*

We downloaded the GWAS Catalog v1.0 from the European Bioinformatics Institute, containing 82,735 curated SNPs.^1^ The catalog was subsequently filtered to exclude SNPs that were classified as intergenic to focus on SNPs associated with transcriptional cis-regulation, resulting 51,892 SNPs. Individual SNPs were processed by the SNPs2ChIP pipeline to determine their enriched phenotype. To validate the robustness of the method, SNPs were grouped by disease and run to determine their combined, enriched phenotype. As the pipeline is designed for high-throughput data analysis, querying thousands of SNPs was done in mere seconds.

## Figures and Tables

**Fig. 4.**
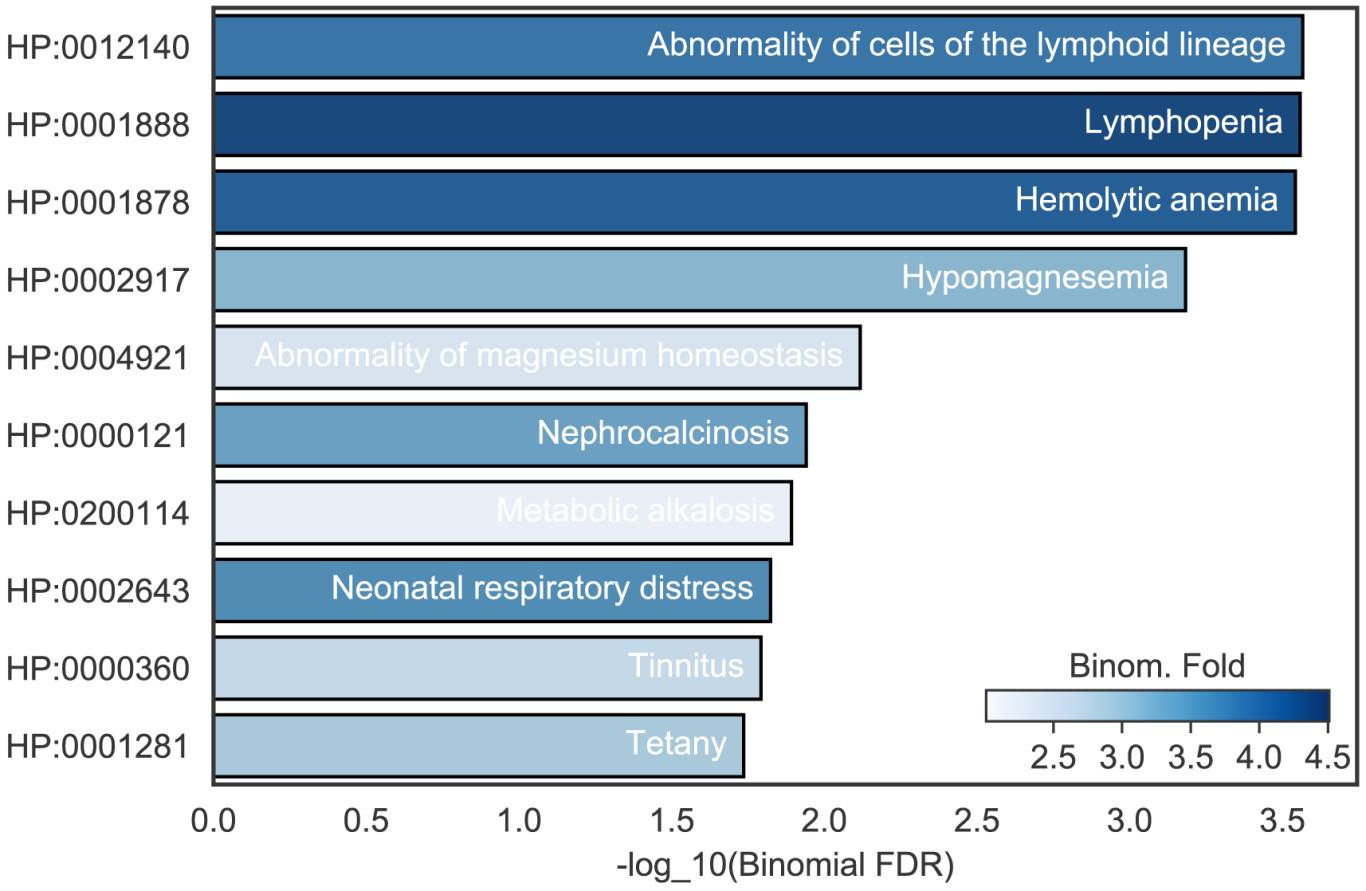
SNPs2ChIP identifies the relevant biological functions given GWAS hits for systemic lupus erythematosus. GREAT binomial FDR and binomial fold for HPO ontology are shown.
